# PPARα activation protects against cholestatic liver injury

**DOI:** 10.1038/s41598-017-10524-6

**Published:** 2017-08-30

**Authors:** Qi Zhao, Rui Yang, Jing Wang, Dan-Dan Hu, Fei Li

**Affiliations:** 1 State Key Laboratory of Phytochemistry and Plant Resources in West China, Kunming Institute of Botany, Chinese Academy of Sciences, Kunming, 650201 China; 2 University of Chinese Academy of Sciences, Beijing, 100049 China; 3 School of Pharmaceutical Science and Yunnan Key Laboratory of Pharmacology of Natural Products, Kunming Medical University, Kunming, 650500 China

## Abstract

Intrahepatic cholestasis induced by drug toxicity, bile salt export pump (BSEP) deficiency, or pregnancy frequently causes cholestatic liver damage, which ultimately may lead to liver fibrosis and cirrhosis. Here, the preventive and therapeutic effects of peroxisome proliferator-activated receptor α (PPARα) signaling activated by fenofibrate was evaluated on cholestatic liver damage. Metabolomic analysis revealed that alpha-naphthyl isothiocyanate (ANIT)-induced intrahepatic cholestasis resulted in the accumulation of serum long-chain acylcarnitines and triglyceride, and the reduced expression of four fatty acid *β*-oxidation (*β*-FAO) relevant genes (Cpt1b, Cpt2, Mcad and Hadha), indicating the disruption of *β-*FAO. The increase of acylcarnitines in hepatic cell resulted in the enhanced expression of anti-oxidative genes glutathione S-transferases (Gsta2 and Gstm3) directly. As direct PPARα-regulated genes, Cpt1b, Cpt2, and Mcad were up-regulated after pretreatment with PPARα agonist, fenofibrate, indicating the improvement of *β-*FAO. In the end, the disrupted bile acid metabolism in the enterohepatic circulation and the enhanced oxidative stress and inflammation cytokines induced by ANIT exposure were significantly recovered with the improvement of *β-*FAO using fenofibrate treatment. These findings provide the rationale for the use of PPARα agonists as therapeutic alternatives for cholestatic liver damage.

## Introduction

Cholestasis that includes primary biliary cirrhosis (PBC), primary sclerosing cholangitis (PSC) and progressive familial intrahepatic cholestasis (PFIC) frequently results in intrahepatic retention of toxic bile acids^1^. If no timely treatment, cholestasis will cause liver fibrosis and cirrhosis^1^. Currently, only two clinic drugs ursodeoxycholic acid (UDCA) and obeticholic acid (OCA) approved by U.S. Food and Drugs Administration (U.S. FDA) are used for the patients who are examined with cholestasis. UDCA could slow the progression of PBC, but up to 40% of patients have inadequate response to its therapy^2^. More clinical case survey is required for OCA because of its limited follow-up data, especially for these patients in the severe stages^2^. Therefore, therapeutic alternatives are urgently needed to consider for these cholestatic patients.

Many mechanism studies on cholestasis development and its therapeutic approaches have been conducted. Recently, using bile salt export pump (BSEP) deficiency-induced cholestasis model revealed that altered fatty acid *β*-oxidation (*β-*FAO) may facilitate cholestatic liver damage^3^. Inhibition of mitochondrial *β*-FAO had been established as a major reason of the hepatotoxicity induced by toxicants and therapeutic agents (e.g. hypoglycin, aspirin, and acetaminophen)^4, 5^. It is well demonstrated that the mitochondrial *β*-FAO is mediated by peroxisome proliferator-activated receptor α (PPARα) that is responsible for lipids homeostasis in the body^6^. One recent review indicated the beneficial actions of PPARα in cholestatic liver disease by summarization of the reports about fibrate and cholestasis^1^. Additionally, one study found that PPARα deficiency will aggravate liver injury in cholic acid (CA)-induced cholestasis^7^. These studies suggest the activation of PPARα signaling might protect against cholestatic liver damage. However, there are limited studies to investigate its protective mechanism.

In the present study, the protective effect of PPARα signaling activated by fenofibrate was evaluated on cholestatic liver damage using ultra-performance chromatography electrospray ionization quadrupole time-of-flight mass spectrometry (UPLC-ESI-QTOFMS)-based metabolomics. MS-based metabolomics has been widely used for identifying the metabolic pathways associated with liver injury^4, 8, 9^, which uncover the potential mechanism of liver injury and its potential therapy. The present study revealed that PPARα signaling activated by fenofibrate could improve mitochondrial *β-*FAO and recover the disorder of bile acid metabolism, and decrease the oxidative stress and inflammation cytokines in alpha-naphthyl isothiocyanate (ANIT)-induced cholestasis, suggesting the potential use of PPARα agonists as therapeutic alternatives for cholestatic liver damage.

## Results

### ANIT-induced cholestasis coupled to impaired mitochondrial *β*-FAO

**Figure 1:**
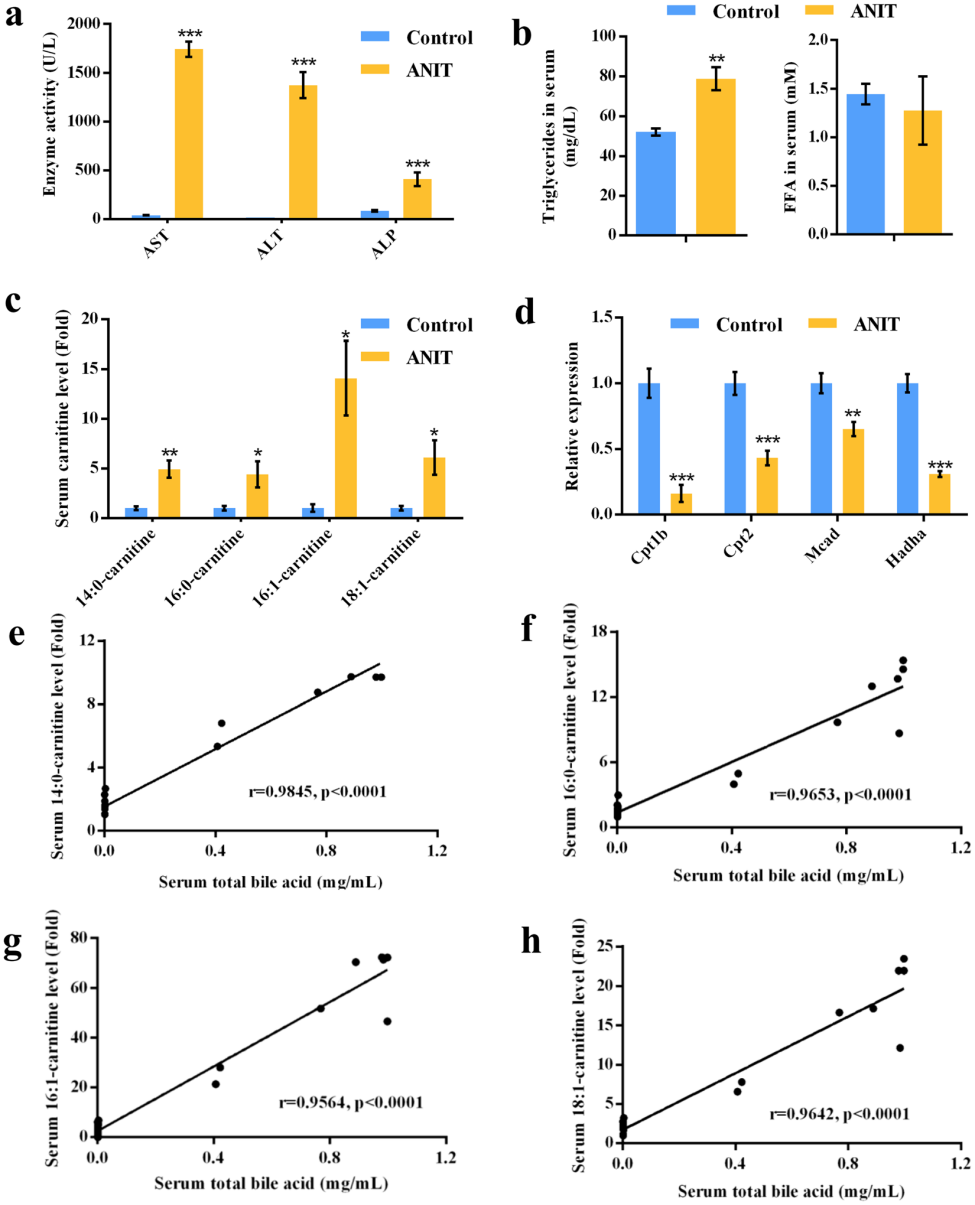
ANIT-induced cholestasis coupled to impaired *β*-FAO in control and ANIT groups. (**a**) Serum AST, ALT, and ALP enzyme activity. (**b**) Serum TG and FFA levels. (**c**) Long-chain acylcarnitine levels. (**d**) QPCR analysis was performed to measure the gene expression of Cpt1b, Cpt2, Mcad and Hadha. Values represented fold change after normalization to control. (**e**) Correlation between the levels of 14:0-carnitine and serum bile acids. (**f**) Correlation between the levels of 16:0-carnitine and serum bile acids. (**g**) Correlation between the levels of 16:1-carnitine and serum bile acids. (**h**) Correlation between the levels of 18:1-carnitine and serum bile acids. Correlation factor (*r*) and *P*-value were estimated with Pearson’s correlation analysis. All data are repressed as mean ± SEM (n = 8). *P < 0.05, **P < 0.01, and ***P < 0.001 verse control.

Biochemical analysis found that ANIT exposure dramatically increased serum aspartate transferase (AST) and alanine transferase (ALT) (*P* < 0.001), as well as serum alkaline phosphatase (ALP) (*P* < 0.001) that is an intrahepatic cholestasis marker (Fig. 1a), indicating a severe cholestasis induced by ANIT. It reported that the levels of triglyceride (TG) and long-chain acylcarnitines, such as myristoylcarnitine (14:0-carnitine), palmitylcarnitine (16:0-carnitine), palmitoleoylcarnitine (16:1-carnitine) and oleoylcarnitine (18:1-carnitine) were increased in serum when *β*-FAO was impaired^3, 4^. In our studies, the level of TG and four long-chain acylcarnitines (*P* < 0.05), 14:0-carnitine, 16:0-carnitine, 16:1-carnitine and 18:1-carnitine was also increased in the serum of ANIT-induced cholestasis (Fig. 1b,c). These acylcarnitines were identified by their MS^2^ fragmentation patterns by comparison with reference compounds (Supplementary Fig. S1). As shown in Supplementary Fig. S1, all these acylcarnitines generated the same fragment ion *m/z* 144^+^. Four mitochondrial *β*-FAO relevant genes, medium-chain acyl-CoA dehydrogenase (Mcad) which catalyzes the first step of FAO^10^, carnitine palmitoyltransferase (Cpt1b and Cpt2) which transports fatty acids across the mitochondrial membrane^10^, and hydroxyacyl-CoA dehydrogenase (Hadha) which catalyzes the final 3 steps in *β*-FAO, were significantly down-regulated (*P* < 0.01) in ANIT group (Fig. 1d). The increased levels of TG and acylcarnitines, and the decreased expression of Cpt1b, Cpt2, Mcad and Hadha indicated the disruption of *β-*FAO. Further analysis revealed that the levels of acylcarnitines were positively correlated with the concentration of serum total bile acid (*P* < 0.0001) (Fig. 1e–h).

### PPARα activation by fenofibrate protected against ANIT-induced liver damage

**Figure 2:**
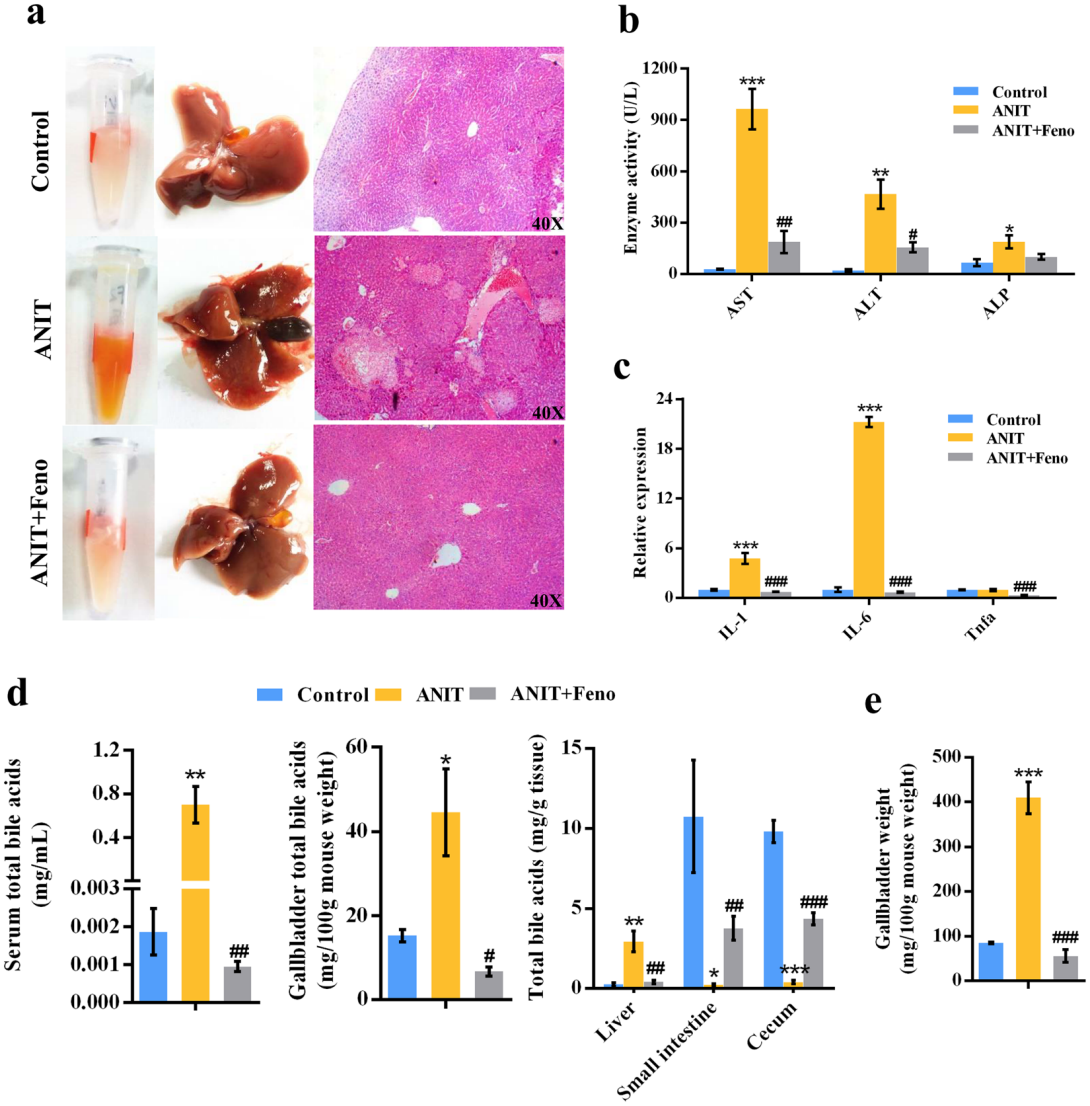
Fenofibrate attenuated ANIT-induced liver injury. (**a**) Phenotype of serum and liver, and H&E staining of liver. (**b**) Serum AST, ALT, and ALP enzyme activity in control, ANIT, and ANIT+Feno groups. (**c**) QPCR analysis of inflammatory factors in liver. Values represented fold change after normalization to control. (**d**) Fenofibrate regulated total bile acids level throughout the enterohepatic circulation. (**e**) Gallbladder weight of control, ANIT, and ANIT+Feno groups mice. All data were repressed as mean ± SEM (n = 5). *P < 0.05, **P < 0.01, and ***P < 0.001 verse control; ^#^ *P* < 0.05, ^##^ *P* < 0.01, and ^###^ *P* < 0.001 verse ANIT.

We assumed that the improvement of *β*-FAO by PPARα agonist^6^ might protect the mice from ANIT-induced cholestasis. As expected, hepatic histological analysis showed that fenofibrate may alleviate parenchymal necrosis, and periportal hemorrhage induced by ANIT (Fig. 2a). Only small lesions were observed in the liver of ANIT and fenofibrate group (ANIT+Feno group), and its histology and serum color were close to the control group (Fig. 2a). Consistent with the histological examination, the elevations of AST, ALT, and ALP in ANIT-induced cholestasis were attenuated by fenofibrate (Fig. 2b). Additionally, fenofibrate treatment recovered the increased inflammatory cytokines interleukin-1 (IL-1) and interleukin-6 (IL-6) in ANIT-induced cholestasis to the normal levels (Fig. 2c). Total bile acid measurement revealed that fenofibrate decreased the elevation of bile acids in the serum, liver and bile induced by ANIT, and was resistant to the decrease of bile acids in small intestine and cecum contents induced by ANIT (*P* < 0.05) (Fig. 2d). Furthermore, gallbladder weight from ANIT group was significantly larger than that from control and ANIT+Feno groups (*P* < 0.001) (Fig. 2e). The ratio of bile acids from liver to intestine was increased to 14.0 in ANIT group, but the ratio was decreased to 0.16 by fenofibrate (Supplementary Fig. S2). These evidences suggested that fenofibrate could attenuate cholestasis induced by ANIT.

Then, another PPARα agonist (bezafibrate) and antagonist (GW6471) treatment were used to evaluate whether fenofibrate play therapeutic role by activating PPARα signaling. Similar with fenofibrate, bezafibrate also reduced ANIT-induced cholestasis, as indicated by liver histology, and lowered AST, ALT, ALP, inflammatory cytokines, acylcarnitines and bile acids (Supplementary Fig. S3). GW6471 diminished the protective effect of fenofibrate, as indicated by liver histology, and increased AST, ALT, ALP, inflammatory cytokines, acylcarnitines and bile acids levels (Supplementary Fig. S4). These evidences suggested that PPARα could attenuate cholestasis induced by ANIT.

### Impaired mitochondrial *β*-FAO was recovered by fenofibrate

**Figure 3:**
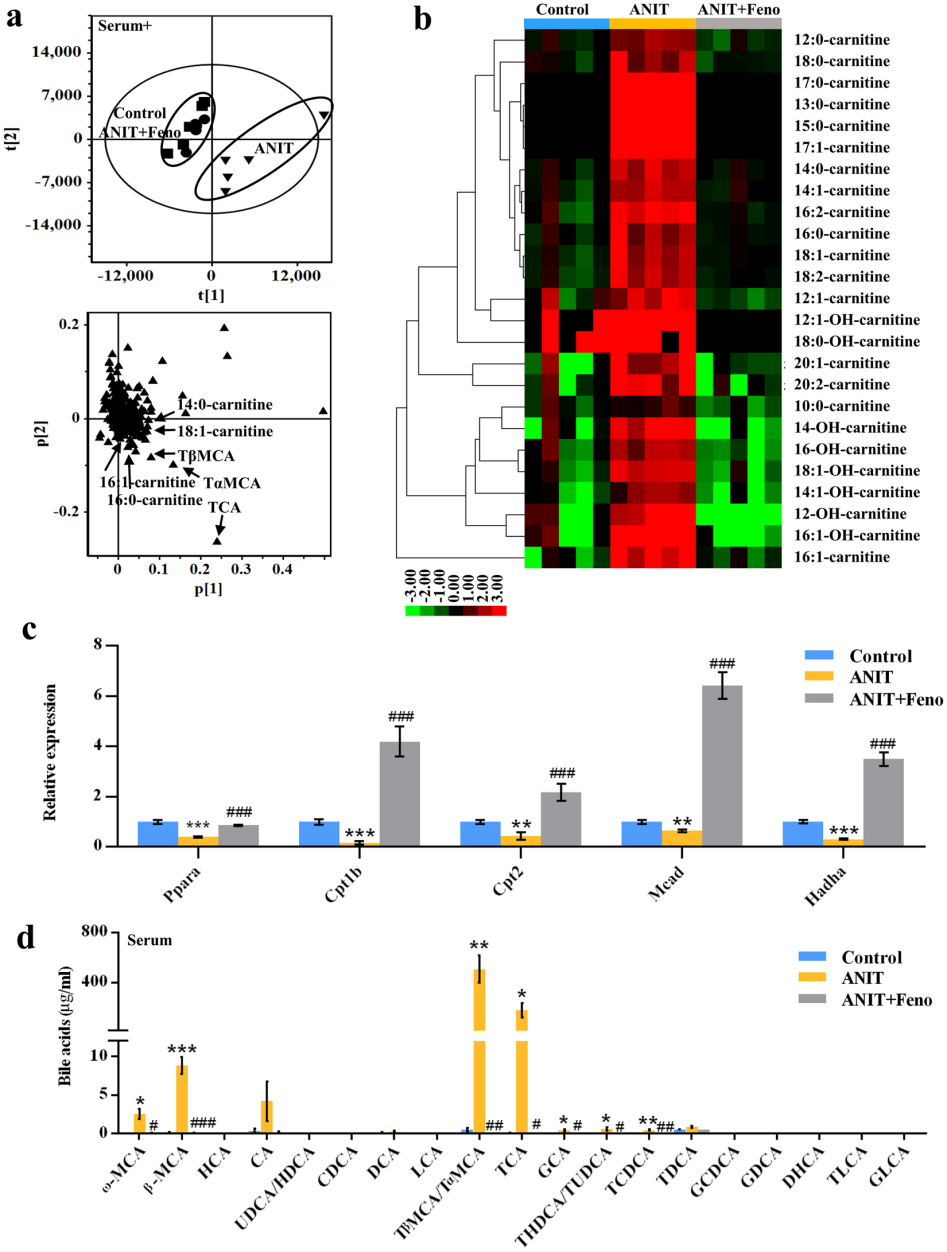
Fenofibrate decreased the accumulation of bile acids and acylcarnitines in serum. (**a**) PCA score plot and loading plot derived from UPLC-QTOFMS data of serum ions. Each point represented an individual mouse serum sample (-top) and an ion in the sample (-bottom). Metabolites were labeled in the loading plot (◾, control group; ▾, ANIT group; ⦁, ANIT+Feno group). (**b**) Heat map analysis of the relative abundance of long-chain carnitines in serum of control, ANIT, and ANIT+Feno groups. The colors on the heatmap corresponded to the contents of long-chain carnitines. Red represented the increase, while green represented the decrease. (**c**) QPCR analysis was performed to measure the gene expression of Pparα and mitochondrial *β*-FAO relevant genes (Cpt1b, Cpt2, Mcad, and Hadha). Values represented fold change after normalization to control. (**d**) Fenofibrate recovered the elevated bile acid levels in serum by ANIT. All data were repressed as mean ± SEM (n = 5). *P < 0.05, **P < 0.01, and ***P < 0.001 verse control; ^#^ *P* < 0.05, ^##^ *P* < 0.01, and ^###^ *P* < 0.001 verse ANIT.

Principal component analysis (PCA) model was used to analyze the serum data sets from control, ANIT, and ANIT+Feno groups. As shown in Fig. 3a, ANIT group was deviated from control and ANIT+Feno groups, indicating that fenofibrate treatment significantly recovered the changed metabolites by ANIT to the normal levels. Four ions 400.3421^+^, 372.3109^+^, 426.3579^+^, and 398.3263^+^ were found to be deviated from the ions cloud in loading scatter plot (Fig. 3a). Chemical formula calculation showed these four ions were corresponded to C_23_H_45_NO_4_, C_21_H_41_NO_4_, C_25_H_47_NO_4_, and C_23_H_43_NO_4_. The MS^2^ fragmentation identified these ions 400.3421^+^ (Rt = 10.39), 372.3109^+^ (Rt = 9.36), 426.3579^+^ (Rt = 10.64), and 398.3263^+^ (Rt = 9.68) as 16:0-carnitine, 14:0-carnitine, 18:1-carnitine, and 16:1-carnitine, respectively (Supplementary Fig. S1). Target metabolomic analysis showed that fenofibrate down-regulated the levels of 25 acylcarnitines that were increased by ANIT (Fig. 3b). Cpt1b, Cpt2, Mcad and Hadha were the mitochondrial *β*-FAO relevant genes regulated by PPARα^6^, so they could be activated by fenofibrate. Quantitative real-time PCR (QPCR) analysis indicated the inhibition of Cpt1b, Cpt2, Mcad and Hadha in cholestasis was increased by fenofibrate (*P* < 0.01) (Fig. 3c). In addition, the concentration of the circulating ketone body β-hydroxybutyrate was slightly increased in the ANIT-induced cholestasis compared to the control group, which was consistent with the results of Abcb11 deficiency-induced cholestasis in mice^3^ (Supplementary Fig. S5). These evidences suggested that fenofibrate could attenuate the impaired *β*-FAO induced by ANIT.

### Disorder of bile acids metabolism was recovered by fenofibrate

**Figure 4:**
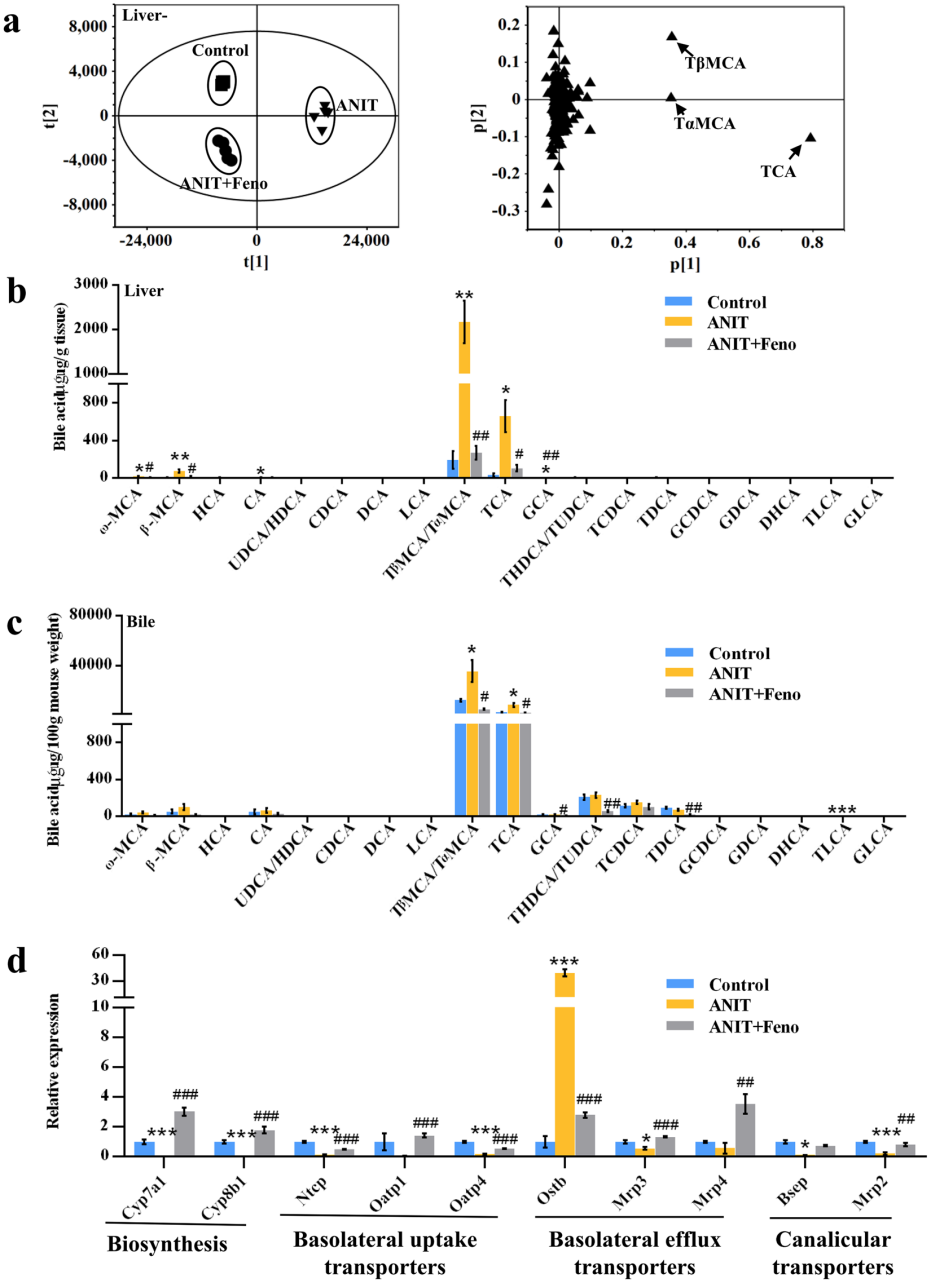
Disrupted bile acid distribution in liver and gallbladder of cholestasis was regulated by fenofibrate. (**a**) PCA score plot and loading plot derived from UPLC-QTOFMS data of hepatic ions. Each point represented an individual mouse liver sample (left) and an ion (right). Metabolites were labeled in the loading plot (◾, control group; ▾, ANIT group; ⦁, ANIT+Feno group). Fenofibrate recovered the elevated bile acid levels in liver (**b**) and gallbladder (**c**) by ANIT. (**d**) QPCR analysis of the hepatic gene expression of bile acid synthesis (Cyp7a1 and Cyp8b1), basolateral uptake transporters (Ntcp, Oatp1 and Oatp4), basolateral efflux transporters (Ostβ, Mrp3 and Mrp4), and canalicular transporters (Bsep and Mrp2). Values represented fold change after normalization to control. All data were repressed as mean ± SEM (n = 5). *P < 0.05, **P < 0.01, and ***P < 0.001 verse control; ^#^ *P* < 0.05, ^##^ *P* < 0.01, and ^###^ *P* < 0.001 verse ANIT.

In the loading scatter plot of PCA, three top increased ions 516.2984^+^, 516.2989^+^, and 516.2987^+^ in the serum of ANIT group were identified as tauro-β-muricholic acid (TβMCA), tauro-α-muricholic acid (TαMCA), and taurocholic acid (TCA), respectively, which were significantly attenuated by fenofibrate (Fig. 3a). Target metabolomics analysis of the levels of individual bile acid in serum was conducted (Fig. 3d). Total 22 bile acids were accurately identified by comparison with reference compounds and quantified based on the standard curves (Supplementary Table S1). As shown in Fig. 3d, the increased levels of ω-muricholic acid (ω-MCA), β-muricholic acid (β-MCA), TβMCA/TαMCA, TCA, glycocholic acid (GCA), taurohyodeoxycholic acid/tauroursodeoxycholic acid (THDCA/TUDCA), and taurochenodeoxycholic acid (TCDCA) in ANIT group were significantly attenuated by fenofibrate (*P* < 0.05) (Fig. 3d). The differences of metabolites in the liver of three groups were also analyzed by PCA model (Fig. 4a), and revealed three top increased ions in the ANIT group, 514.2845^−^, 514.2843^−^, and 514.2847^−^ which was recovered by fenofibrate (Fig. 4a). The MS^2^ fragmentation demonstrated that the ions 514.2843^−^ (Rt = 6.33, 6.42, and 7.19 min) were [M − H]^−^ of TβMCA, TαMCA, and TCA, respectively. Further target metabolomic analysis showed that the increased levels of ω-MCA, β-MCA, TβMCA/TαMCA, TCA, and GCA in liver of ANIT exposure were recovered by fenofibrate (*P* < 0.05) (Fig. 4b). Similarly, the levels of TβMCA/TαMCA, TCA, and taurolithocholic acid (TLCA) were increased significantly in bile with ANIT treatment (*P* < 0.05) (Fig. 4c). After fenofibrate treatment, the levels of TβMCA/TαMCA, TCA, GCA, THDCA/TUDCA, and taurodeoxycholic acid (TDCA) were decreased in bile (*P* < 0.05) (Fig. 4c). Furthermore, the expression level of hepatic genes involved in bile acid synthesis and transport were measured by QPCR (Fig. 4d). The levels of two bile acid synthesis genes, cholesterol 7α-hydroxylase (Cyp7a1) and sterol 12α-hydroxylase (Cyp8b1), decreased in ANIT group^11^ were recovered by fenofibrate (Fig. 4d). The expressions of three basolateral uptake transporters, sodium taurocholate cotransporting polypeptide (Ntcp), and organic anion transporting polypeptides Oatp1^11^ and Oatp4 were significantly decreased in ANIT group, but the levels of these genes were increased by fenofibrate (Fig. 4d). Three basolateral efflux transporters organic solute transporter β (Ostβ), multidrug resistance proteins Mrp3 and Mrp4, and two canalicular transporters Bsep and Mrp2 were also analyzed among three groups (Fig. 4d). The expression level of Ostβ was increased 40-fold by ANIT compared with control group^11^, which was attenuated by fenofibrate (Fig. 4d). The decreased expression levels of Mrp2, Mrp3, Mrp4, and Bsep by ANIT were recovered by fenofibrate (Fig. 4d).

**Figure 5:**
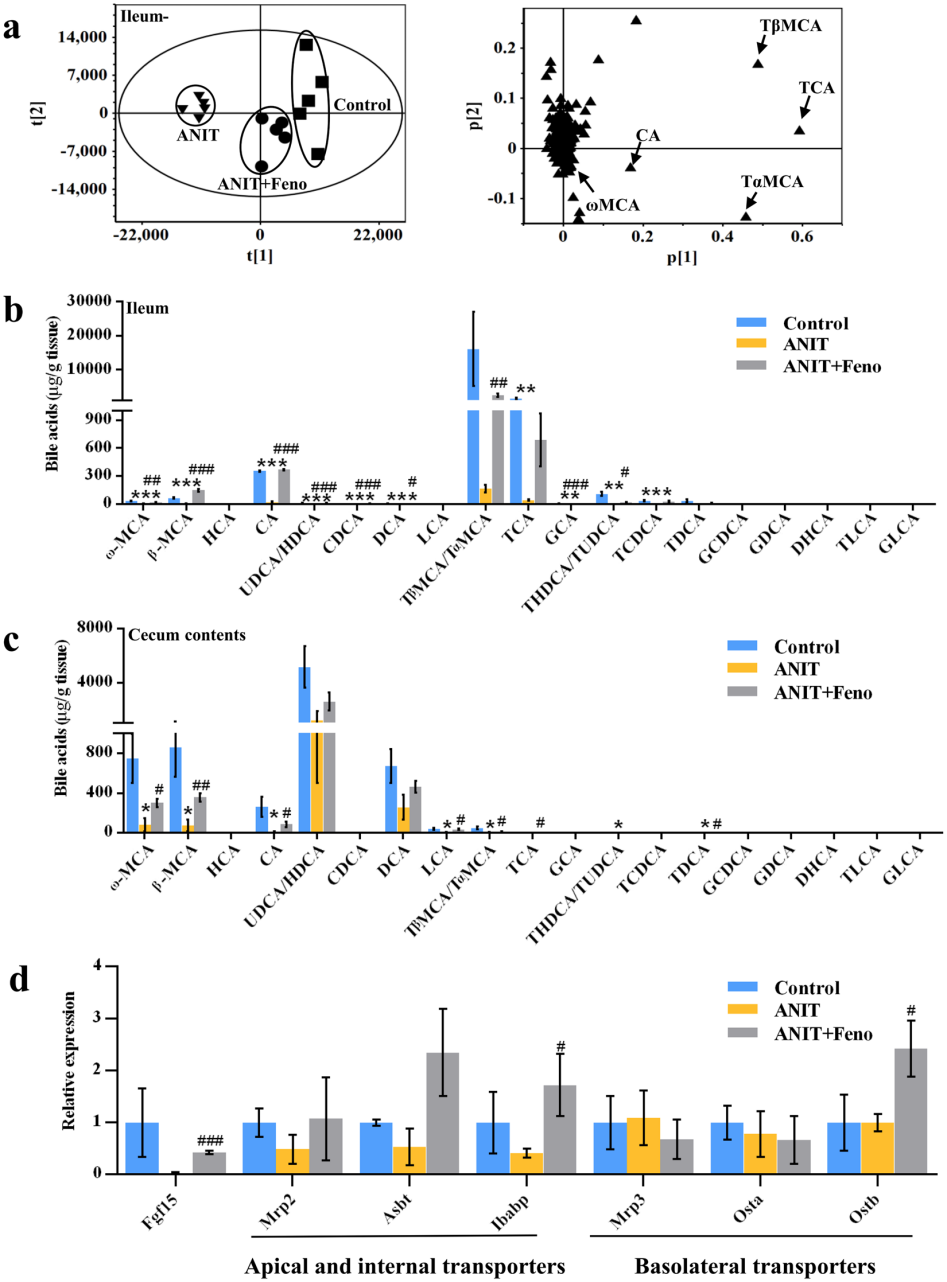
Bile acids distribution in intestine of cholestasis was recovered by fenofibrate. (**a**) PCA score plot and loading plot derived from UPLC-QTOFMS data of ileal ions. Each point represented an individual mouse ileum sample (left) and an ion (right). Metabolites were labeled in the loading plot (◾, control group; ▾, ANIT group; ⦁, ANIT+Feno group). Fenofibrate recovered the reduced bile acid levels in ileum (**b**) and cecum (**c**) by ANIT. (**d**) QPCR analysis was performed to measure the ileum gene expression of Fgf15, apical and internal transporters (Mrp2, Asbt and Ibabp), and basolateral transporters (Mrp3, Ostα and Ostβ). Values represented fold change after normalization to control. All data were repressed as mean ± SEM (n = 5). *P < 0.05, **P < 0.01, and ***P < 0.001 verse control; ^#^ *P* < 0.05, ^##^ *P* < 0.01, and ^###^ *P* < 0.001 verse ANIT.

Intestinal metabolomics was used to determine the differences among control, ANIT, and ANIT+Feno groups. As shown in Fig. 5a, five top decreased ions 514.2846^−^, 514.2848^−^, 514.2843^−^, 407.2803^−^, and 407.2849^−^ in the ileum of ANIT group were recovered by fenofibrate. These ions 514.2846^−^ (Rt = 6.33), 514.2848^−^ (Rt = 6.42), 514.2843^−^ (Rt = 7.19), 407.2803^−^ (Rt = 8.61), and 407.2849^−^ (Rt = 7.67) were identified as TβMCA, TαMCA, TCA, CA, and ω-MCA based on the chemical formula calculation and MS^2^ fragmentation. Further target analysis showed the reduced bile acids by ANIT in both small intestine and cecum were recovered by fenofibrate (Fig. 5b and c). The decreased levels of ω-MCA, β-MCA, CA, ursodeoxycholic acid/hyodeoxycholic acid (UDCA/HDCA), chenodeoxycholic acid (CDCA), deoxycholic acid (DCA), GCA, and THDCA/TUDCA in ileum of ANIT group were recovered with fenofibrate (*P* < 0.05) (Fig. 5b). The changed trends of bile acids in duodenum and jejunum were similar to that of the ileum (Supplementary Fig. S6). The decreased levels of ω-MCA, β-MCA, CA, lithocholic acid (LCA), TβMCA/TαMCA, and TDCA in cecum contents by ANIT were recovered with fenofibrate administration (*P* < 0.05) (Fig. 5c). As bile acids were reabsorbed (>95%) mainly in the ileum^12^, the expression levels of ileum apical transporters Mrp2, apical sodium dependent bile acid transporter (Asbt), and ileal bile acid binding protein (Ibabp) that moved bile acids from small intestine across the apical brush border membrane, and basolateral transporters (Mrp3, Ostα, and Ostβ) that made the bile acids shuttle to the basolateral membrane and efflux into the portal circulation, were also evaluated using QPCR. Fenofibrate increased the expression levels of fibroblast growth factor 15 (Fgf15), Ibabp, and Ostβ (*P* < 0.05) (Fig. 5d). Overall, PPARα signaling activated by fenofibrate could recover the distribution of bile acids in enterohepatic circulation via regulating the genes associated with bile acids synthesis and transport.

### Oxidative stress in cholestasis was eliminated by fenofibrate

**Figure 6:**
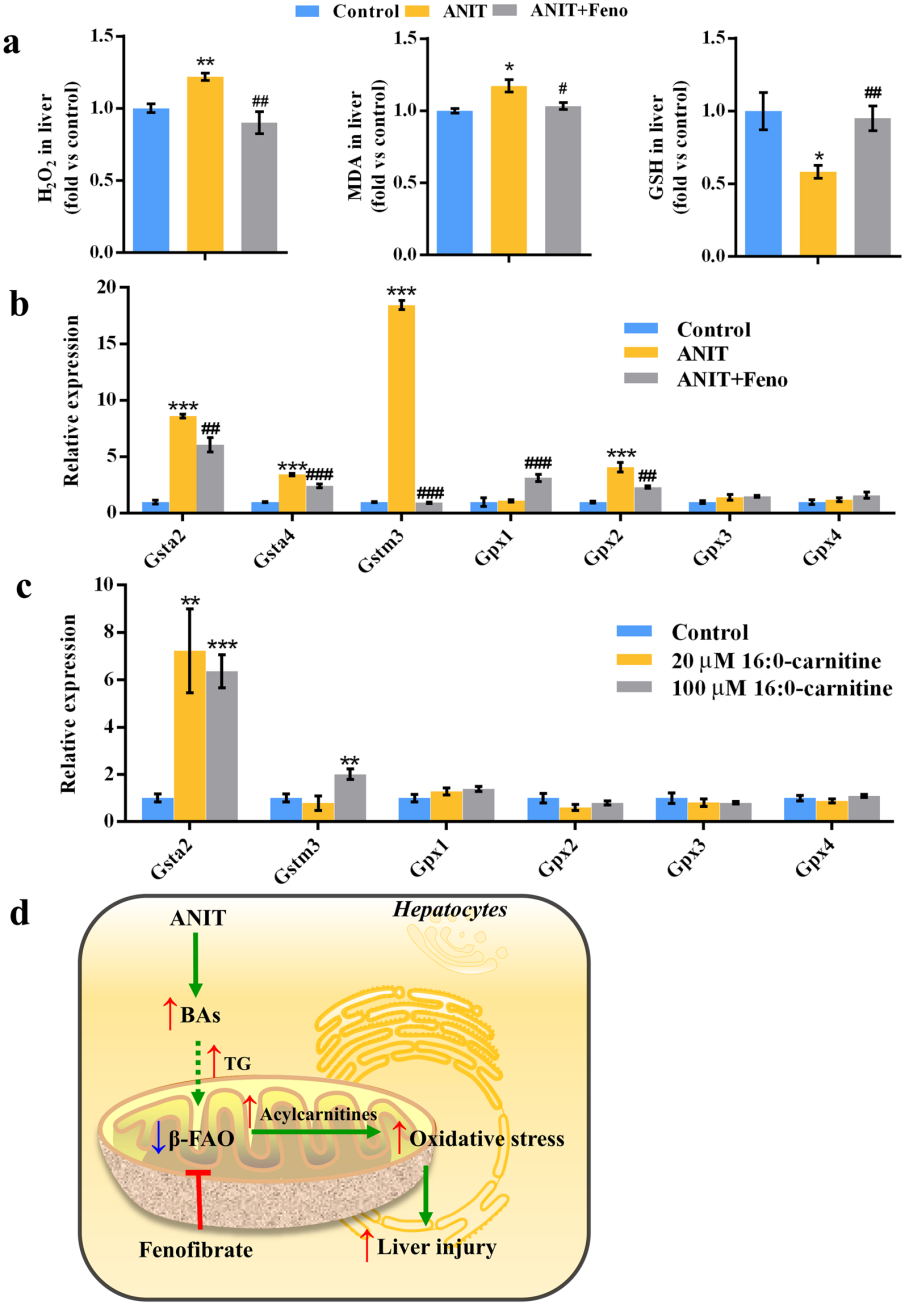
Fenofibrate eliminated oxidative stress and attenuated cholestatic liver injury. (**a**) Hepatic H_2_O_2_, MDA, and GSH levels in control, ANIT, and ANIT+Feno groups. (**b**) QPCR analysis of the gene expression of hepatic Gst and Gpx isofroms. (**c**) QPCR analysis of the gene expression of Gst and Gpx isofroms in HepG2 cells. Values represented fold change after normalization to control. All data were repressed as mean ± SEM (n = 5). *P < 0.05, **P < 0.05, and ***P < 0.001 verse control; ^#^ *P* < 0.05, ^##^ *P* < 0.01, and ^###^ *P* < 0.001 verse ANIT. (**d**) Schematic model of the therapeutic effects from fenofibrate treatment. ANIT resulted in the disruption of *β*-FAO. Fenofibrate treatment decreased the levels of acylcarnitines that caused intracellular oxidative stress in the liver by activating *β*-FAO relevant genes (Cpt1b, Cpt2, Mcad, and Hadha). Finally, the cholestatic liver injury was alleviated via the decrease of oxidative stress.

It has been demonstrated that increased serum acylcarnitines are specific for mitochondrial dysfunction, which could induce oxidative stress^13, 14^. We hypothesized that the increase of a series of acylcarnitines in ANIT-induced cholestasis generated oxidative stress. It was found hepatic and serum H_2_O_2_ and malondialdehyde (MDA) was increased (*P* < 0.05) and hepatic glutathione (GSH) level was depleted in ANIT group (*P* < 0.05) (Fig. 6a and Supplementary Fig. S7). The expression levels of several anti-oxidative genes were up-regulated in ANIT group, including glutathione *S*-transferases (Gsta2, Gsta4, and Gstm3) and glutathione peroxidase 2 (Gpx2) (*P* < 0.001) (Fig. 6b). These evidences demonstrated the greater oxidative stress in ANIT-induced cholestasis. The expression levels of some anti-oxidative genes, including Gsta2 and Gstm3, were also up-regulated after HepG2 cells exposure to 16:0-carnitine, indicating the accumulation of acylcarnitines generated the oxidative stress (Fig. 6c). After ANIT-induced cholestasis was treated with fenofibrate, the levels of Gsta2, Gsta4, Gstm3, and Gpx2 were significantly decreased (*P* < 0.01) (Fig. 6b). The hepatic H_2_O_2_, MDA, and GSH levels were also turned back to the normal levels (*P* < 0.05) (Fig. 6a). Additionally, fenofibrate treatment alone cannot directly affect GSH levels (Supplementary Fig. S8). These findings indicated that ANIT-induced oxidative stress could be eliminated by fenofibrate.

### Therapeutic effect of fenofibrate against ANIT-induced cholestasis

**Figure 7:**
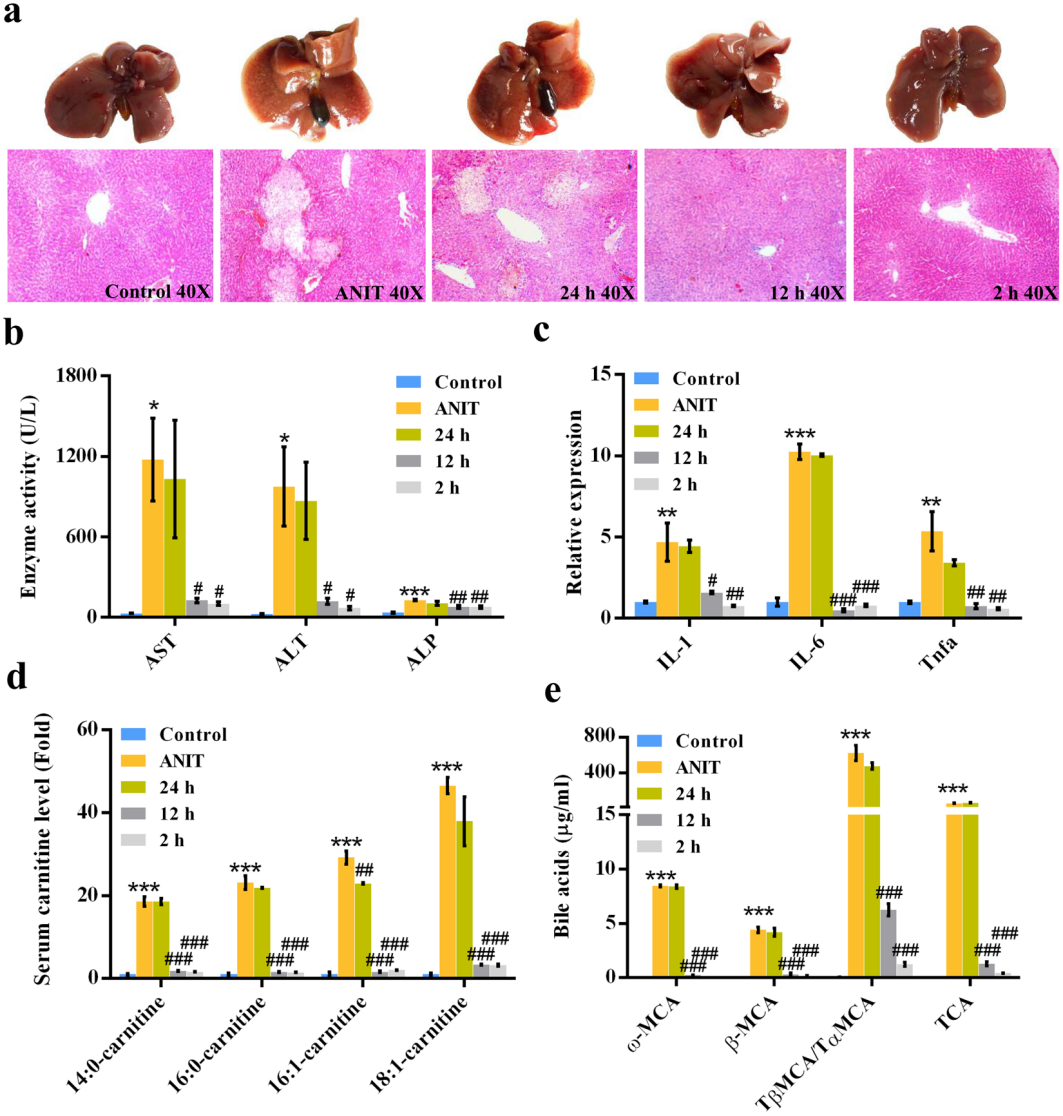
Therapeutic effect of fenofibrate against ANIT-induced cholestasis in control, ANIT, 24 h, 12 h and 2 h groups. (**a**) Phenotype and H&E staining of liver. (**b**) Serum AST, ALT, and ALP enzyme activity. (**c**) QPCR analysis of inflammatory factors in liver. Values represented fold change after normalization to control. (**d**) Long-chain acylcarnitine levels. (**e**) Bile acids levels. All data were repressed as mean ± SEM (n = 5). *P < 0.05, **P < 0.01, and ***P < 0.001 verse control; ^#^ *P* < 0.05, ^##^ *P* < 0.01, and ^###^ *P* < 0.001 verse ANIT.

Biochemical analysis of the therapeutic effect of fenofibrate against ANIT-induced cholestasis was conducted (Fig. 7). Hepatic histological analysis showed that only small lesions were observed in the liver of 12 h and 2 h groups (Fig. 7a). The elevations of AST, ALT, ALP, inflammatory cytokines, acylcarnitines, and bile acids in ANIT-induced cholestasis were also attenuated in 12 h and 2 h groups (Figs. 7b–e). No remarkable therapeutic effect of fenofibrate was observed in 24 h after ANIT treatment (Fig. 7). These findings suggested that PPARα agonist might play a therapeutic effect against early ANIT-induced cholestasis (0–12 h), but it showed limit therapeutic effect against late ANIT-induced cholestasis, such as 24 h after ANIT treatment.

## Discussion

It has been demonstrated that MS-based metabolomics is a powerful tool to determine the potential mechanism of liver diseases through its influence on endogenous metabolites^15^. Lyso-phosphocholines (LPCs) and bile acids are the common changed biomarkers for the different types of liver injury^16, 17^, including liver fibrosis, cirrhosis and chemical-induced cholestasis. ANIT is a hepatotoxicant used in mice and rats to model human drug-induced intrahepatic cholestasis. One recent study found that two LPCs were increased in serum of ANIT-induced mice cholestasis and demonstrated the role of lipid-regulated NF-κB/IL-6/STAT3 axis in ANIT-induced liver injury^18^. In the present study, using metabolomics revealed that a series of long-chain acylcarnitines were increased in the serum of ANIT-induced cholestasis model indicated the impaired *β*-FAO. *In vitro* assay demonstrated that long-chain acylcarnitines may increase liver oxidative stress and inflammation. PPARα activation by clinical drug fenofibrate may decrease the accumulation of acylcarnitines and the impaired *β*-FAO, and repair liver injury. Consistent with this study, BSEP deficiency-induced cholestasis also could impair *β*-FAO which increased urinary excretion of four glycine-conjugated metabolites and TG in the liver^3^. These data indicate that PPARα signaling which regulates *β*-FAO may be one potential target against cholestatic liver damage.

One study in 1993 found that the PPARα agonist bezafibrate was beneficial for patients with cholestasis^19^. Then, some pilot studies demonstrated the efficacy of fenofibrate and UDCA combination in patients with PBC who failed to response to UDCA^20–25^. Combination of fenofibrate and UDCA could decrease serum AST, ALT, ALP, γ-glutamyl transpeptidase, and TG in PBC patients who are not response to UDCA alone^23^. In this study, we found that fenofibrate could protect against ANIT-induced liver injury in mice. It could decrease the value of serum AST, ALT and improve the impaired liver histology by ANIT exposure. Previous studies reported the protective role of fibrates in ethinylestradiol and chlorpromazine-induced intrahepatic cholestasis and bile duct-ligated-induced extrahepatic cholestasis^26, 27^. One recent study found that another PPARα agonist Wy-14643 also could prohibit from ANIT-induced cholestasis^18^. Here, metabolomic analysis revealed that the abnormal distribution of bile acids in the serum, liver, gallbladder and intestine of ANIT-induced cholestasis could be recovered by fenofibrate. Gene analysis showed that fenofibrate up-regulated the reduced expression levels of Cyp7a1, Cyp8b1, Ntcp, Oatp1, Oatp4, Mrp3, Mrp4, Bsep, and Mrp2 in liver and the reduced Fgf15 and Ibabp in ileum by ANIT exposure. More importantly, the accumulation of long-chain acylcarnitines in the serum was decreased accordingly, and the inhibited expression of PPARα target genes was promoted following by fenofibrate treatment, including Cpt1b, Cpt2, and Mcad. These evidences suggest that PPARα activation could protect against cholestatic liver injury via the regulation of *β*-FAO, which finally recover bile acids metabolism in the enterohepatic circulation.

Currently, UDCA and OCA are the only two FDA-approved drugs for the treatment of cholestasis^2^, and both of them are the ligands of farnesoid X receptor (FXR). It is well demonstrated that FXR is the crucial nuclear receptor, which is responsible for the homeostasis of bile acids. In addition to FXR, PPARα also could directly regulate some genes involved in bile acids metabolism, including Cyp7a1, Cyp8b1, and Asbt^28^. For example, the expression levels of bile acid biosynthesis Cyp7a1 and Cyp8b1 were up-regulated in mice liver following by PPARα activation, and this effect would be diminished in PPARα-null mice^29^. The expression of human CYP7A1 genes could be up-regulated by PPARα agonist fatty acids and Wy-14643 treatment^30^, and human ASBT were identified as direct positive PPARα-regulated genes through a functional DR-1 motif present in its promoter^31^. In contrast to the above data, another study observed PPARα reduced the expression of Cyp7a1 in the mice^32^. Additionally, we found that PPARα-knockout mice were more sensitive to CA-induced cholestasis than wild-type mice^7^, confirming the role of PPARα in regulation of metabolic pathways of bile acids. However, the mechanism by which PPARα mediates the metabolism and transport of bile acids need further studies to clarify.

H_2_O_2_ and MDA levels in the mice liver and serum was increased in ANIT-induced cholestasis, indicating the elevation of oxidative stress. Because of the elevation of oxidative stress, we hypothesized that the transcription factor, NF-E2-related factor 2 (Nrf2) a sensor of oxidative stress, would be activated^33, 34^. Nrf2 activation can be revealed by up-regulated of a series of anti-oxidative stress genes, including glutathione *S*-transferases and superoxide dismutases. In our study, the expression levels of Gsta2, Gsta4, Gstm3 and Gpx2 were increased in the ANIT-induced cholestasis. These findings indicate greater oxidative stress in ANIT-induced cholestasis. HepG2 cells may be a useful model system for the study of *β*-FAO and oxidative stress^35, 36^. Previous study found that HepG2 cells treated with 16:0-carnitine had an IC_50_ of 76 µM, illustrating the negative effect of acylcarnitine accumulation in liver cells^37^. *In vitro* assay indicated that the increase of long-chain acylcarnitines could result in the oxidative stress through elevating the expression of Gsta2 and Gstm3. The hepatic H_2_O_2_ and MDA was recovered to normal levels by fenofibrate treatment which decreased the levels of Nrf2 target genes Gsta2, Gsta4, Gstm3 and Gpx2. Consistent with the decreased ROS, fenofibrate recovered the levels of hepatic GSH that was depleted in ANIT-induced cholestasis. These evidences demonstrated that PPARα activation by fenofibrate could protect against cholestatic liver damage.

In conclusion, this present study demonstrated the protective role of PPARα signaling activated by fenofibrate in ANIT-induced cholestasis (Fig. 6d). It is likely that PPARα activation ameliorates cholestatic liver damage through its regulation of *β*-FAO genes, including Cpt1b, Cpt2, and Mcad. The beneficial actions of PPARα activation in the liver will ultimately lead to the decrease of the inflammation cytokines, which could be extrapolated to other cholestatic conditions (e.g. drugs- or Bsep inhibition-induced). These findings support the view that PPARα agonists may be used as therapeutic alternatives for cholestatic liver damage by upregulating *β*-FAO.

## Methods

### Chemicals and reagents

Fenofibrate and bezafibrate was obtained from the National Institute for the Control of Pharmaceutical and Biological Products (Beijing, China). GW6471, ANIT, formic acid, chlorpropamide, lauroylcarnitine (12:0-carnitine), myristoylcarnitine (14:0-carnitine), palmitoylcarnitine (16:0-carnitine), stearoylcarnitine (18:0-carnitine), CA, UDCA, HDCA, CDCA, DCA, TCA, GCA, THDCA, TCDCA, TDCA, TLCA, glycochendeoxycholic acid (GCDCA), glycodeoxycholic acid (GDCA), dehydrocholic acid (DHCA), and glycolithocholic acid (GLCA) were purchased from Sigma-Aldrich (St. Louis, Missouri, USA). Hyocholic acid (HCA), TβMCA, and ωMCA were purchased from Santa Cruz Biotechnology, Inc. (Dallas, TX). TUDCA and LCA was purchased from Medchemexpress (Monmouth Junction, NJ, USA). TαMCA and βMCA were purchased from Steraloids (Newport, RI). All solvents and organic reagents were of the highest grade commercially available.

### Animals

Male C57BL/6 J mice (6- to 8-week-old) were obtained from Shanghai laboratory animal center (SLAC), China. These mice were maintained under a standard 12 h light/12 h dark cycle with relative humidity at 60–70%. The institutional animal care and use committee at the Kunming Institute of Botany, Chinese Academy of Sciences approved all the produces involving mice and the experiments were carried out in accordance with the approved guidelines and regulations. (I) Oral gavage of a single dose of ANIT (75 mg/kg dissolved in corn oil) to mice was used to prepare cholestasis model (n = 8) in Fig. 1. (II) To investigate the preventive effect of fenofibrate in ANIT-induced toxicity, the mice were randomly assigned into four groups (n = 5): (1) control group; (2) fenofibrate group; (3) ANIT group; (4) ANIT+Feno group. Fenofibrate and ANIT+Feno groups were treated with fenofibrate (200 mg/kg dissolved in 0.5% sodium carboxymethylcellulose (CMC-Na)) for 5 consecutive days. After fenofibrate was treated for three days, ANIT and ANIT+Feno groups mice were given a single oral dose of ANIT (75 mg/kg). (III) To investigate the role of other PPARα agonist, bezafibrate, in ANIT-induced cholestasis, the mice were randomly assigned into four groups (n = 5): (1) control group; (2) ANIT group; (3) ANIT and bezafibrate group (ANIT + Beza group); (4) ANIT+Feno group. ANIT + Beza and ANIT+Feno groups were treated with bezafibrate and fenofibrate (200 mg/kg) for 5 consecutive days, respectively. After bezafibrate and fenofibrate was treated for three days, ANIT, ANIT + Beza, and ANIT+Feno groups mice were given a single oral dose of ANIT (75 mg/kg). (IV)To investigate the role of PPARα antagonist, GW6471, in ANIT-induced cholestasis, the mice were randomly assigned into four groups (n = 5): (1) control group; (2) ANIT group; (3) ANIT+Feno + GW6471 group; (4) ANIT+Feno group. ANIT+Feno group was treated with fenofibrate (200 mg/kg) for 5 consecutive days. ANIT+Feno + GW6471 group was cotreated with GW6471 (20 mg/kg, intraperitoneal administration, 30 min before fenofibrate) and fenofibrate for 5 consecutive days. After fenofibrate was treated for three days, ANIT, ANIT+Feno + GW6471, and ANIT+Feno groups mice were given a single oral dose of ANIT (75 mg/kg). (V) To investigate the therapeutic effect of fenofibrate in ANIT-induced cholestasis, the mice were randomly assigned into five groups (n = 5): (1) control group; (2) ANIT group; (3) 24 h group; (4) 12 h group; (5) 2 h group. ANIT, 24 h, 12 h, 2 h groups mice were given a single oral dose of ANIT (75 mg/kg). Then, 24 h group was treated with fenofibrate (200 mg/kg) 24 h and 36 h after ANIT treatment. 12 h group was treated with fenofibrate 12 h, 24 h and 36 h after ANIT treatment. 2 h group was treated with fenofibrate 2 h, 24 h and 36 h after ANIT treatment. All these mice were killed after ANIT administration for 48 h. Plasma, liver, bile, small intestine (duodenum, jejunum, ileum, and ileal mucosal), and cecum contents were harvested and frozen at −80 °C before analysis. All the collections were performed on 4 h-fasted mice to get bile juice.

### Biochemical assay and histological examination

AST, ALT, and ALP activities, as well as free fatty acids (FFAs) and TG concentration were measured using the method described previously^3^. H_2_O_2_, MDA, and β-hydroxybutyrate was measured by assay kits (Nanjing Jiancheng Bioengineering Institute, Nanjing City, China). Fresh liver samples were fixed in 10% buffered formalin for 24 h. After dehydration in different concentrations of alcohol and xylene, the tissue was embedded in paraffin. Five-micrometer serial sections were made and stained with hematoxylin and eosin (H&E). Histological findings were examined by light microscopy.

### Sample preparation

Plasma and bile samples were prepared using the method described previously^7^. Liver and intestine samples were prepared as the following procedure. 100 mg liver or 50 mg small intestine samples were minced in 1.0 ml of 50% aqueous acetonitrile and shaken for 20 min at room temperature. After centrifugation at 15000 rpm for 20 min, 100 µl of supernatant was transferred to a new Eppendorf vial and diluted with 100 µl of 100% acetonitrile. After the mixture was centrifuged at 15000 rpm for 20 min, a 5 µl aliquot was injected into the LC-MS system. 20 mg of cecum contents were mixed with 200 µl of 50% aqueous acetonitrile. After shaking at room temperature for 20 min, the samples were centrifuged at 15000 rpm for 20 min to obtain cecum content extract supernatants. Subsequently, 100 µl of supernatant was transferred to a new Eppendorf vial and diluted with 200 µl of 50% aqueous acetonitrile. A 5 µl aliquot was injected into the LC-MS system after centrifugation at 15000 rpm for 20 min. 5 µM chlorpropamide was used as the internal standard.

### UPLC-ESI-QTOFMS analysis

The liquid chromatography system consisted of a 1290 Autosampler, Quat Pump, and Photodiode Array Detector (Agilent, Santa Clara, CA). The endogenous metabolites were separated via a XDB-C18 column (2.1 × 100 mm, 1.8 µM). The mobile phase comprised 0.01% formic acid solution (A) and acetonitrile containing 0.01% formic acid solution (B). The flow rate was set at 0.3 ml/min with a gradient ranging from 2% to 98% acetonitrile (B) in 16 min run. Column temperature was set at 45 °C. The mass signals were collected in both positive and negative mode on the Agilent 6530 Q-TOFMS (Agilent, Santa Clara, CA, USA), which was operated in full-scan mode at *m/z* 100 to 800. Capillary voltage was set at 3.5 kV. Nitrogen was applied as both collision gas and drying gas (9 l/min). The nebulizer pressure was 35 psi, and the drying gas temperature was set at 350 °C.

### Multivariate data analysis

Raw data from UPLC-ESI-QTOFMS system were processed using Mass Profinder and Mass Profiler Professional software (Agilent, Santa Clara, CA, USA), which generated a data matrix consisting of peak areas corresponding to a unique Rt and *m/z*. The data set was further exported into SIMCA-P+13.0 software (Umetrics, Kinnelon, NJ, USA) for PCA. PCA analyses were performed with mean center scaling, and showed in the form of the score plot and loading plot. Clustering of a serious of serum long-chain carnitines was performed using Cluster and TreeView software, which was visualized using heat map^38^.

### Gene expression analysis

Total RNA was extracted from approximately 100 mg liver, and 10 mg ileal mucosa using TRIzol reagent (Lifetechnologies, Carlsbad, CA). QPCR was carried out using SYBR green PCR master mix (TaKaRa, Dalian, China) in a CFX Connect Real-Time System (Bio-Rad Laboratories). QPCR primer sequences and the abbreviation of genes are shown in Supplementary Tables S2 and S3. Target mRNA levels were normalized to those of *β*-actin mRNA and expressed as fold change relative to the control group. Thermal cycling conditions were as follows: 3 min at 95 °C, followed by 40 cycles of 95 °C for 10 s, 55 °C for 30 s and 72 °C for 40 s. All the measurements were performed in triplicates.

### Cell culture

HepG2 (ATCC HB-8065) was maintained in 1640 containing 10% fetal bovine serum (FBS) and 100 U penicillin/streptomycin. Cells were seeded to 6 well plate at a density of 0.8 × 10^6^ cells per well. Subsequently, adherent cells were exposed with 16:0-carnitine (20 and 100 µM) for 16 h. The total RNA was extracted as described above.

### Data analysis

The data were presented as the means ± SEM. Statistical analysis was performed using the one-way ANOVA or the two-tailed Student *t*-test. *P-*values of less than 0.05 were considered significant. Correlation factor (r) was estimated with Pearson’s correlation analysis.

### Data availability

The datasets generated during the current study are available from the corresponding author on reasonable request.

## Electronic supplementary material

Supplementary Information
