# Supplementary material for: PPARα activation protects against cholestatic liver injury
Source: Sci Rep. 2017 Aug 30;7:9967. doi: 10.1038/s41598-017-10524-6 (PMC5577315; doi:10.1038/s41598-017-10524-6)
Supplement: Supplementary file 1 — Supplementary Information [file 41598_2017_10524_MOESM1_ESM.pdf]

SUPPORTING INFORMATION

PPARα activation protects against cholestatic liver injury

Qi Zhao[1,2], Rui Yang[1,2], Jing Wang[1], Dan-Dan Hu[1,3], and Fei Li[1,*]

[1]State Key Laboratory of Phytochemistry and Plant Resources in West China, Kunming Institute of Botany, Chinese Academy of Sciences, Kunming 650201, China

[2]University of Chinese Academy of Sciences, Beijing 100049, China

[3]School of Pharmaceutical Science and Yunnan Key Laboratory of Pharmacology of Natural Products, Kunming Medical University, Kunming 650500, China

Correspondence and requests for materials should be addressed to F.L. (email: lifeib@mail.kib.ac.cn)

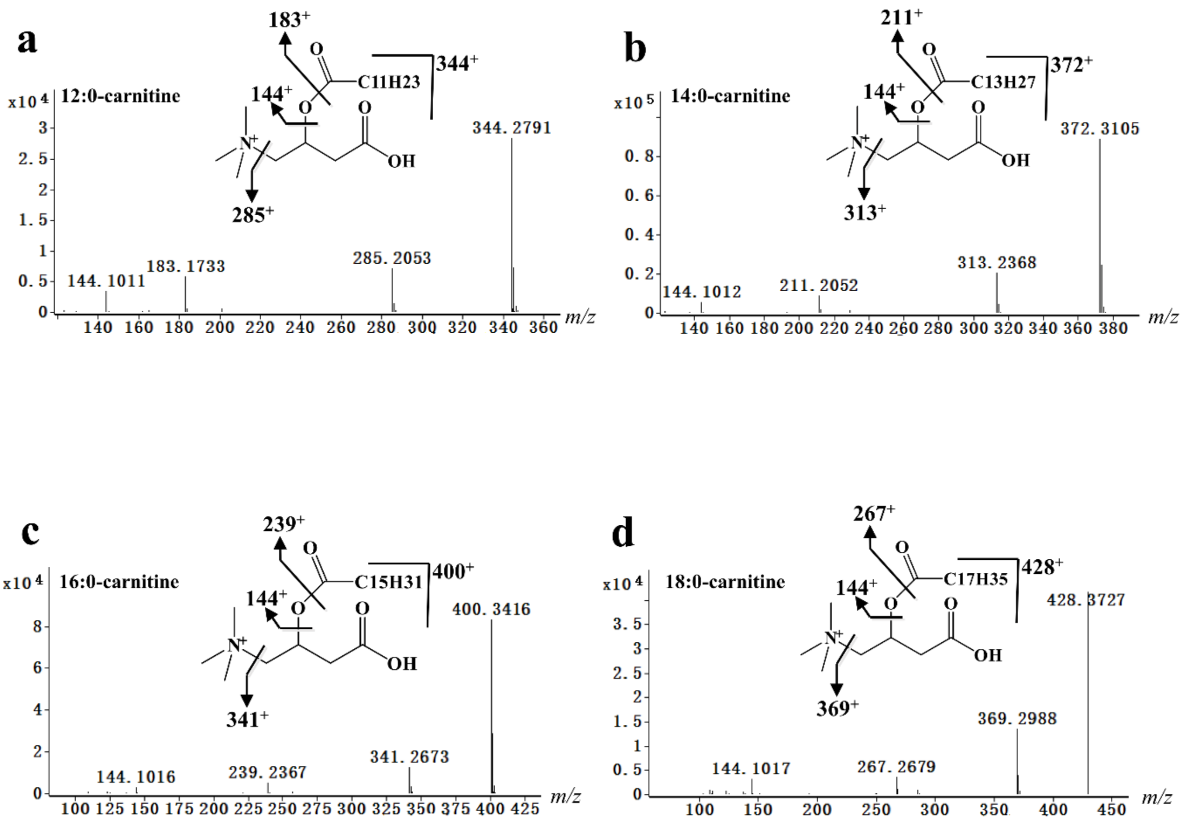

Supplementary Fig. S1. Tandem MS of 12:0-carnitine (a), 14:0-carnitine (b), 16:0-carnitine (c), and 18:0-carnitine (d). MS^2 fragmentation was conducted with collision energy at 10 eV.

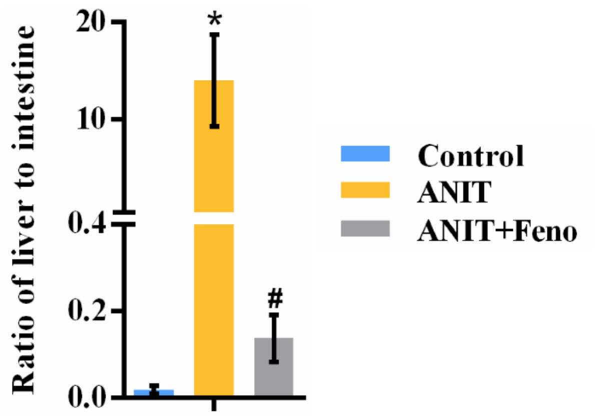

Supplementary Fig. S2. Improvement of bile acids accumulation in the liver of ANIT group by fenofibrate. Relative bile acid pool size=hepatic total bile acid/small intestine total bile acid. All data were repressed as mean ± SEM (n=5). **P*<0.05 verse control; #*P*<0.05 verse ANIT.

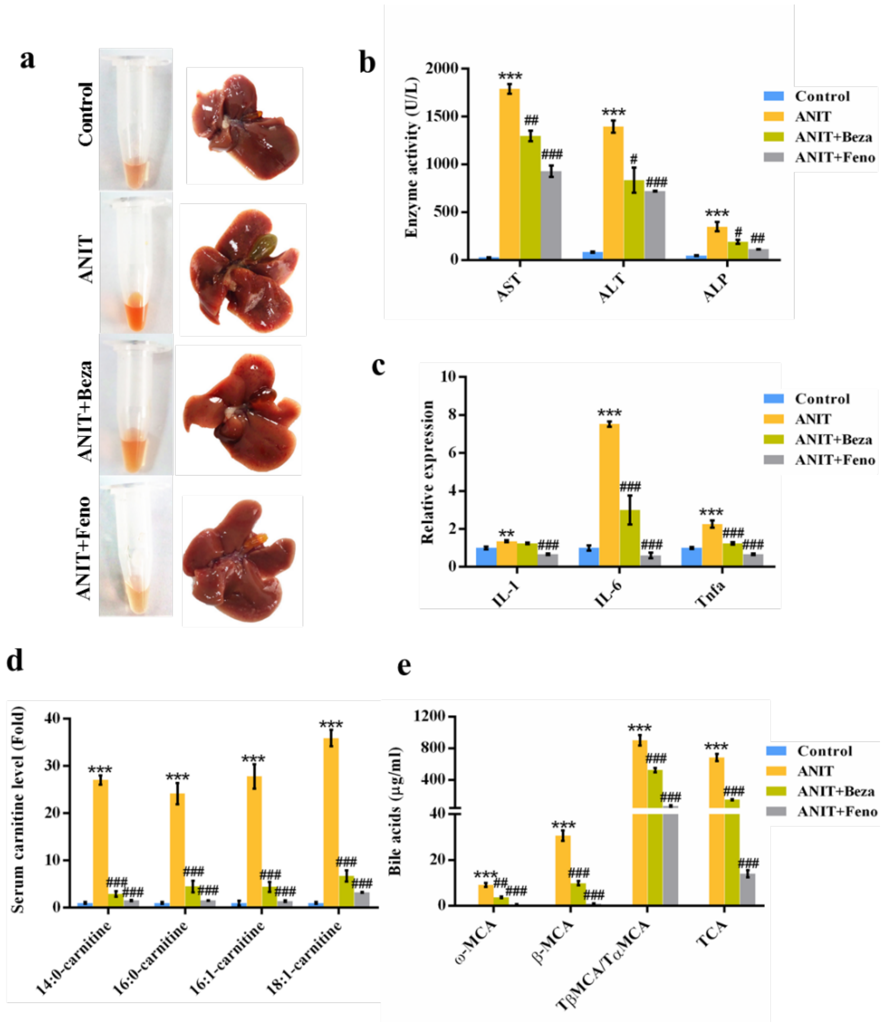

Supplementary Fig. S3. Bezafibrate attenuated ANIT-induced liver injury. (a) Phenotype of liver and serum. (b) Serum AST, ALT, and ALP enzyme activity in control, ANIT, ANIT+Beza, and ANIT+Feno groups. (c) QPCR analysis of inflammatory factors in liver. Values represented fold change after normalization to control. (d) Bezafibrate decreased the accumulation of acylcarnitines. (e) Bezafibrate decreased the accumulation of bile acids. All data were repressed as mean ± SEM (n=5). $^{**}P<0.01$, and $^{***}P<0.001$ verse control; $^{\#}P<0.05$, $^{\#\#}P<0.01$, and $^{\#\#\#}P<0.001$ verse ANIT.

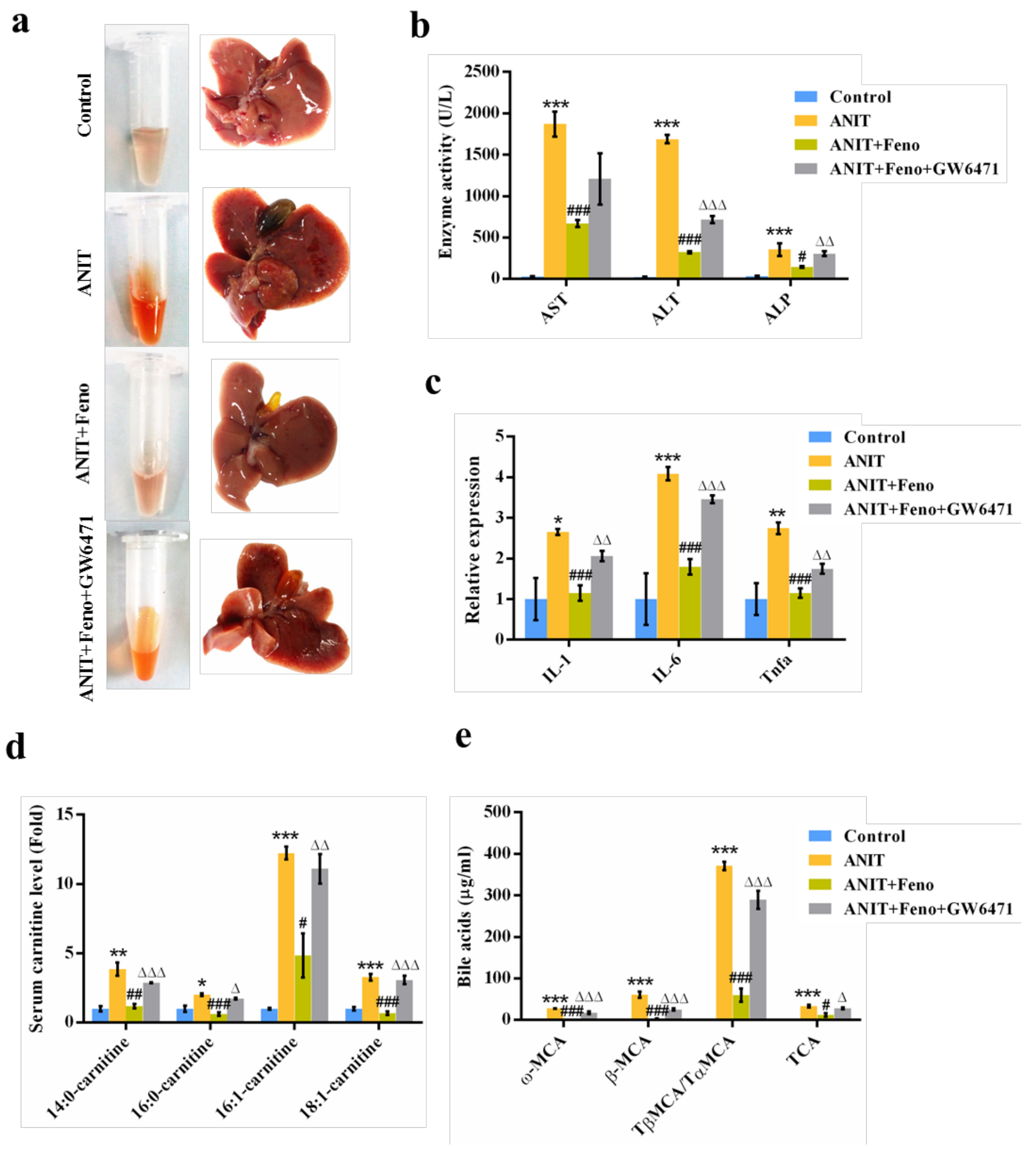

Supplementary Fig. S4. GW6471 inhibited the protective effect of fenofibrate. (a) Phenotype of liver and serum. (b) Serum AST, ALT, and ALP enzyme activity in control, ANIT, ANIT+Feno and ANIT+Feno+GW6471 groups. (c) QPCR analysis of inflammatory factors in liver. Values represented fold change after normalization to control. (d) GW6471 increased the accumulation of acylcarnitines compare with ANIT+Feno groups. (e) GW6471 increased the accumulation of bile acids compared with ANIT+Feno groups. All data were repressed as mean ± SEM (n=5). **P*<0.05, ***P*<0.01, and ****P*<0.001 verse control; $^{\#}P$<0.05, $^{\#\#}P$<0.01, and $^{\#\#\#}P$<0.001 verse ANIT; $^{\Delta}P$<0.05, $^{\Delta\Delta}P$<0.01, and $^{\Delta\Delta\Delta}P$<0.001 verse ANIT+Feno.

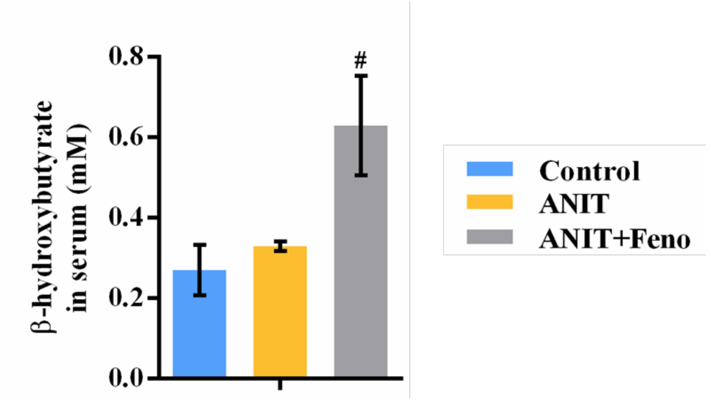

Supplementary Fig. S5. Serum was used for β-hydroxybutyrate measurement. All data were repressed as mean ± SEM (n=5). $^{\#}P$<0.05 verse ANIT.

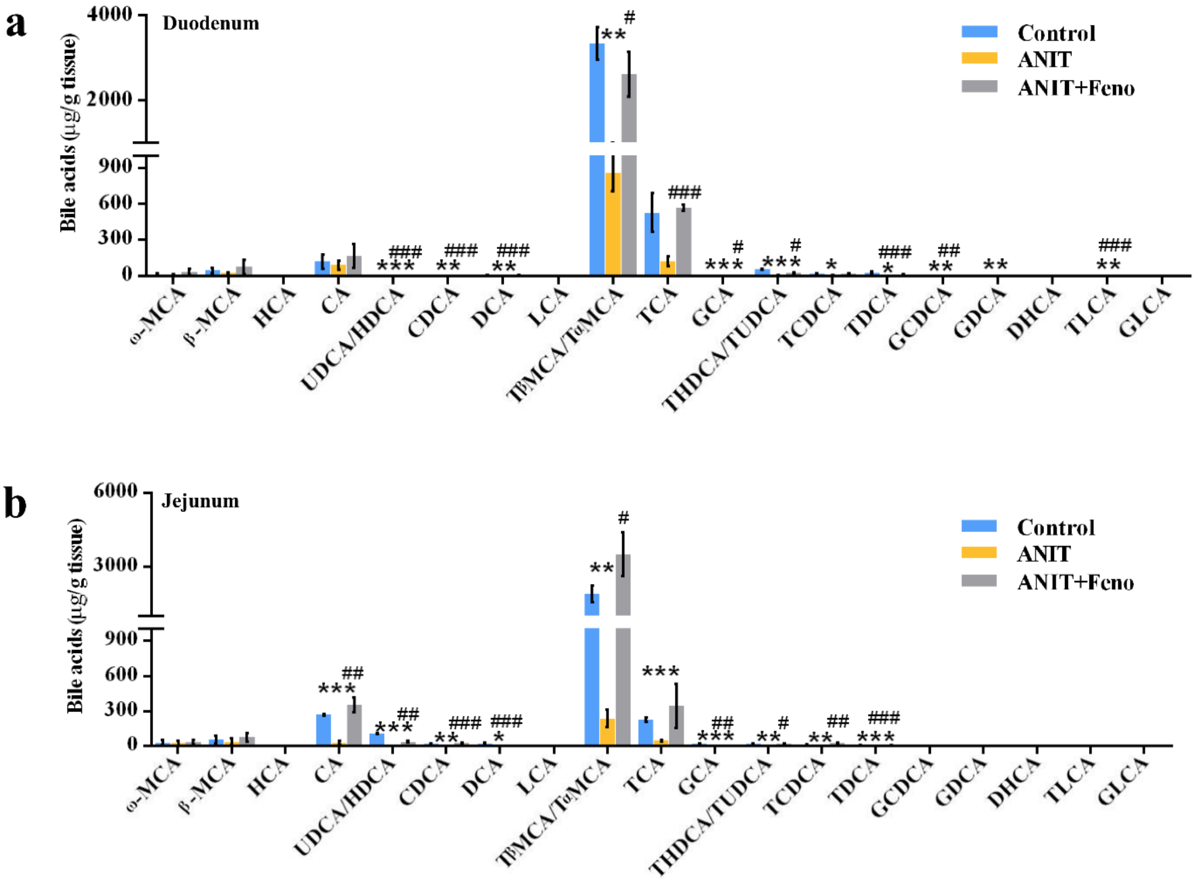

Supplementary Fig. S6. Fenofibrate recovered the reduced bile acid levels in duodenum (a) and jejunum (b) caused by ANIT. All data were repressed as mean ± SEM (n=5). **P*<0.05, ***P*<0.01, and ****P*<0.001 verse control; $^{\#}P$<0.05, $^{\#\#}P$<0.01, and $^{\#\#\#}P$<0.001 verse ANIT.

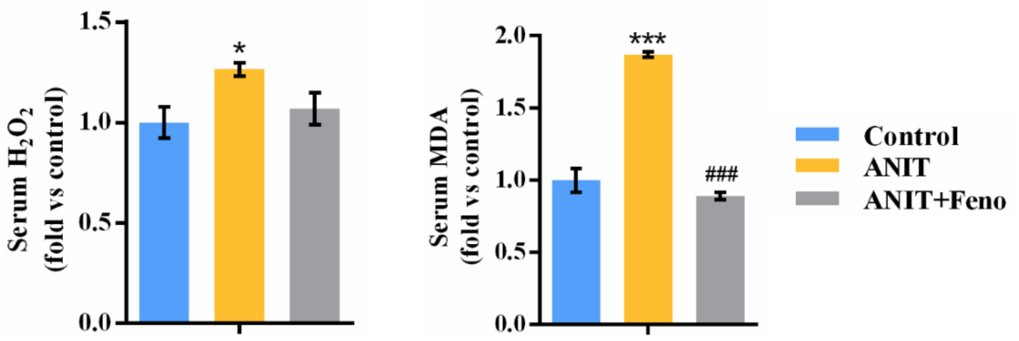

Supplementary Fig. S7. Serum H_2O_2 and MDA levels in control, ANIT, and ANIT+Feno groups. All data were repressed as mean ± SEM (n=5). *P<0.05, and ***P<0.001 verse control; $^{###}P$<0.001 verse ANIT.

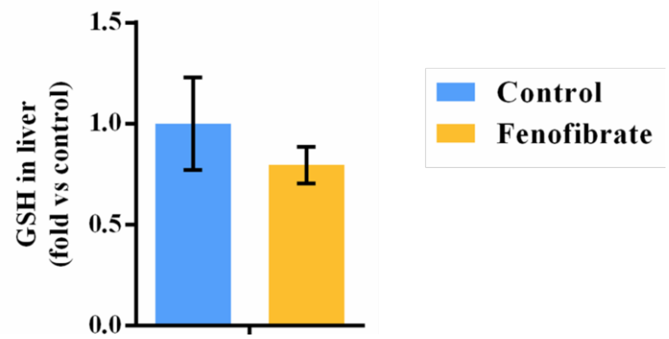

Supplementary Fig. S8. Hepatic GSH contents were measurement between control- and fenofibrate-treated mice without ANIT. All data were repressed as mean ± SEM (n=5).

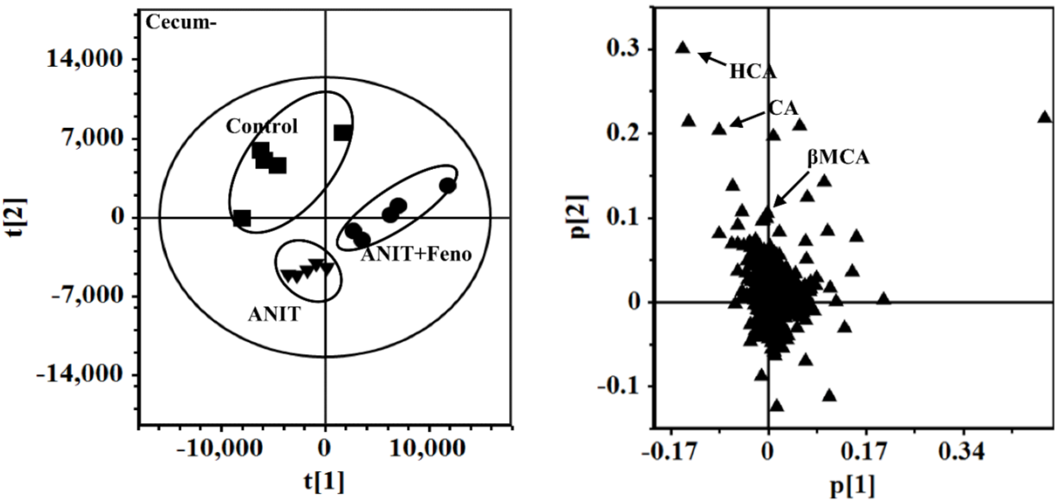

Supplementary Fig. S9. PCA score plot and loading plot derived from LC-MS data of cecum ions. Each point represented an individual mouse sample (left) and an ion (right). Metabolites were labeled in the loading plot (■, control-treated mice in score plot; ▼, ANIT-treated mice in score plot; ●, ANIT+Feno-treated mice in score plot).

Supplementary Table S1. Parameters of regression for the measurement of bile acids.

Compounds	Rt (min)	Regression equation	Correlation coefficient	Linear range (ng/mL)
ω-MCA	7.675	y=6993x+8205	0.9994	10-4000
β-MCA	7.958	y=7933x+34005	0.9995	10-15000
HCA	8.264	y=2694x+9790	0.9999	10-50
CA	8.601	y=77793x+478912	0.9991	10-12000
UDCA/HDCA	8.853	y=5816x+84410	1.0000	10-50
CDCA	10.042	y=7097x+64272	0.9998	10-50
DCA	10.210	y=103771x+704302	0.9999	10-500
LCA	12.028	y=11624x+109732	0.9994	10-50
TβMCA/TαMCA	6.336	y=138810x+685632	0.9665	10-800000
TCA	7.197	y=721096x+3000000	0.9995	10-400000
GCA	7.591	y=118897x+1000000	0.9995	10-700
THDCA/TUDCA	7.104	y=643652x+348574	0.9991	10-2000
TCDCA	8.092	y=630057x+2000000	0.9998	10-500
TDCA	8.394	y=1000000x-55686	0.9992	10-2000
GCDCA	8.642	y=141373x+835292	0.9999	10-50
GDCA	8.859	y=168337x+676712	0.9993	10-50
DHCA	7.825	y=34245x+263626	0.9992	10-50
TLCA	9.676	y=564727x+12864	0.9997	10-50
GLCA	10.310	y=183670x+990037	0.9995	10-50

Supplementary Table S2. Primer sequences for qRT-PCR.

Gene	Abbreviation	Sequence
Acyl-CoA oxidase 1	*Acox1*	CCGCCACCTTCAATCCAGAG
		CAAGTTCTCGATTTCTCGACGG
Ileal bile acid transporter	*Asbt*	TGGTGTAGACGAAGAGGCAA
		GCCTATTGGATAGATGGCGA
Bile salt export pump (Abcb11)	*Bsep*	CCAGAACATGACAAACGGAA
		AAGGACAGCCACACCAACTC
Carnitine palmitoyltransferase 1	*Cpt 1b*	CCTCTCATGGTGAACAGCAA
		GGTCCAGTTTACGGCGATAC
Carnitine palmitoyltransferase 2	*Cpt 2*	CAGCACAGCATCGTACCCA
		TCCCAATGCCGTTCTCAAAAT
Cholesterol 7α-hydroxylase	*Cyp7a1*	GGGAATGCCATTTACTTGGA
		GTCCGGATATTCAAGGATGC
Sterol 12α-hydroxylase	*Cyp8b1*	TCCTCAGGGTGGTACAGGAG
		GATAGGGGAAGAGAGCCACC
Fibroblast growth factor 15	*Fgf15*	GCCATCAAGGACGTCAGCA
		CTTCCTCCGAGTAGCGAATCAG
Glutathione S-transferase α2	*Gsta2*	TTATGTCCCCCAGACCAAAG
		CCTGTTGCCCACAAGGTAGT
Glutathione S-transferase α4	*Gsta4*	AGACCACGGAGAGGCT
		CCTGACCACCTCAACATAGGG
Glutathione S-transferase mu 3	*Gstm3*	CCCCAACTTTGACCGAAGC
		GGTGTCCATAACTTGGTTCTCCA
Glutathione peroxidase 1	*Gpx1*	TGGACTGGTGGTGCTCG
		CGTCACTGGGTGTTGGC
Glutathione peroxidase 2	*Gpx2*	GGGCTGTGCTGATTGAGA
		CGGACATACTTGAGGCTGTT
Glutathione peroxidase 3	*Gpx3*	GGCTTCCCTTCCAACC
		AATTTCTGCTCTTTCTCCC
Glutathione peroxidase 4	*Gpx4*	ACGATGCCCACCCACT
		CCACGCAGCCGTTCTT
Hydroxyacyl-CoA dehydrogenase	*Hadha*	AAGGGGATGTGGCAGTTATT
		ACTCCTGATTTGGTCGTTGG
Ileal bile acid-binding protein	*Ibabp*	GGTCTTCCAGGAGACGTGAT
		ACATTCTTTGCCAATGGTGA
Interleukin-1	*IL-1*	CCCTGCAGCTGGAGAGTGTGGA
		TGTGCTCTGCTTGTGAGGTGCTG

Supplementary Table S2. (Continued)

Gene	Abbreviation	Sequence
Interleukin-6	*IL-6*	TGATGCACTTGCAGAAAACA
		ACCAGAGGAAATTTTCAATAGGC
Multidrug resistance protein (Abcc2)	*Mrp2*	TCCAGGACCAAGAGATTTGC
		TCTGTGAGTGCAAGAGACAGGT
Multidrug resistance protein (Abcc3)	*Mrp3*	CTGGGTCCCCTGCATCTAC
		GCCGTCTTGAGCCTGGATAAC
Multidrug resistance protein (Abcc4)	*Mrp4*	AGCTTCAACGGTACTGGGATA
		TCGTCGGGGTCATACTTCTC
Medium-chain acyl-CoA dehydrogenase	*Mcad*	GCGAGCAGAAATGAAACTCC
		AGCTCTAGACGAAGCCACGA
Sodium taurocholate contransporting polypeptide	*Ntcp*	AGGGGGACATGAACCTCAG
		TCCGTCGTAGATTCCTTTGC
Organic anion transporting protein 1	*Oatp1*	ACTCCCATAATGCCCTTGG
		TAATCGGGCCAACAATCTTC
Organic anion transporting protein 4	*Oatp4*	ACCAAACTCAGCATCCAAGC
		TAGCTGAATGAGAGGGCTGC
Organic solute transporter α	*Ostα*	CACTGGCTCAGTTGCCATTT
		GCATACGGCATAAAACGAGGT
Organic solute transporter β	*Ostβ*	GTATTTTCGTGCAGAAGATGCG
		TTTCTGTTTGCCAGGATGCTC
Peroxisome proliferator-activated receptor α	*Pparα*	CCCAAGGGAGGAATAGCTTCT
		CTCTGCGATGCGGTTCCAA
Tumour necrosis factor α	*Tnfα*	CCACCACGCTCTTCTGTCTAC
		AGGGTCTGGGCCATAGAACT

Supplementary Table S3. Abbreviations.

	**Abbreviations**	**Full manes**
1	ALP	Alkaline phosphatase
2	ALT	Alanine transferase
3	ANIT	Alpha-naphthyl isothiocyanate
4	ANIT+Beza group	ANIT and bezafibrate group
5	ANIT+Feno group	ANIT and fenofibrate group
6	Asbt	Apical sodium dependent bile acid transporter
7	AST	Aspartate transferase
8	BSEP	Bile salt export pump
9	CA	Cholic acid
10	CDCA	Chenodeoxycholic acid
11	CMC-Na	Sodium carboxymethylcellulose
12	Cpt1b	Carnitine palmitoyltransferase 1b
13	Cpt2	Carnitine palmitoyltransferase 2
14	Cyp7a1	Cholesterol 7α-hydroxylase
15	Cyp8b1	Sterol 12α-hydroxylase
16	DCA	Deoxycholic acid
17	DHCA	Dehydrocholic acid
18	FBS	Fetal bovine serum
19	FDA	Food and Drugs Administration
20	FFA	Free fatty acid
21	Fgf15	Fibroblast growth factor 15
22	FXR	Farnesoid X receptor
23	GCA	Glycocholic acid
24	GCDCA	Glycochendeoxycholic acid
25	GDCA	Glycodeoxycholic acid
26	GLCA	Glycolithocholic acid
27	Gpx	Glutathione peroxidase
28	GSH	Glutathione
29	Gsta	Glutathione S-transferase
30	Hadha	Hydroxyacyl-CoA dehydrogenase
31	HCA	Hyocholic acid
32	HDCA	Hyodeoxycholic acid
33	H&E	Hematoxylin and eosin
34	Ibabp	Ileal bile acid binding protein
35	IL-1	Interleukin-1
36	IL-6	Interleukin-6
37	LCA	Lithocholic acid
38	LPC	Lyso-phosphocholine
39	Mcad	Medium-chain acyl-CoA dehydrogenase
40	MDA	Malondialdehyde
41	Nrf2	NF-E2-related factor 2

42	Ntcp	Sodium taurocholate cotransporting polypeptide
43	Oatp1	Organic anion transporting polypeptide 1
44	Oatp4	Organic anion transporting polypeptide 4
45	OCA	Obeticholic acid
46	Ostβ	Organic solute transporter β
47	PBC	Primary biliary cirrhosis
48	PCA	Principal component analysis
49	PFIC	Progressive familial intrahepatic cholestasis
50	PPARα	Peroxisome proliferator-activated receptor α
51	PSC	Primary sclerosing cholangitis
52	QPCR	Quantitative real-time PCR
53	SLAC	Shanghai laboratory animal center
54	TCA	Taurocholic acid
55	TCDCA	Taurochenodeoxycholic acid
56	TDCA	Taurodeoxycholic acid
57	TG	Triglyceride
58	THDCA	Taurohyodeoxycholic acid
59	TLCA	Taurolithocholic acid
60	TUDCA	Tauroursodeoxycholic acid
61	TαMCA	Tauro-α-muricholic acid
62	TβMCA	Tauro-β-muricholic acid
63	UDCA	Ursodeoxycholic acid
64	UPLC-ESI-QTOFMS	Ultra-performance chromatography electrospray ionization quadrupole time-of-flight mass spectrometry
65	β-FAO	Fatty acid β-oxidation
66	β-MCA	β-muricholic acid
67	ω-MCA	ω-muricholic acid
68	12:0-carnitine	Lauroylcarnitine
69	14:0-carnitine	Myristoylcarnitine
70	16:0-carnitine	Palmitylcarnitine
71	16:1-carnitine	Palmitoleoylcarnitine
72	18:0-carnitine	Stearoylcarnitine
73	18:1-carnitine	Oleoylcarnitine
